# Skin Dryness and Pruritus in Older Adults With and Without Heart Failure and Implications for Home Care Nursing

**DOI:** 10.7759/cureus.92770

**Published:** 2025-09-20

**Authors:** Yumi Fukuyama, Chie Furushima

**Affiliations:** 1 Institute of Nursing, Faculty of Medicine, Saga University, Saga, JPN

**Keywords:** geriatrics, heart failure, home care, pruritus, sebum deficiency, skin barrier

## Abstract

Background: Pruritus is a common but under-recognized symptom in patients with heart failure (HF), particularly among older adults. While systemic factors have been implicated, the role of local skin conditions remains unclear.

Objective: The objective of this study is to investigate the prevalence and severity of pruritus in older adults with HF compared to older adults without HF and identify local skin parameters associated with pruritus.

Methods: We conducted a cross-sectional study including older adults with HF and non-HF controls. Skin assessments were conducted on the forearm and lower leg, including measurements of moisture, sebum content, and pH. Pruritus was evaluated using a visual analog scale (VAS). Multivariate logistic regression was performed to determine factors independently associated with pruritus.

Results: A total of 100 older adults (≥65 years) were included in the analysis (HF: n=74; non-HF: n=26). There were no differences in age, sex, BMI, or occupation. Compared with the non-HF group, the HF group had significantly lower skin moisture on both the forearm and the lower leg (p<0.001), although this may have been influenced by measurement conditions. Pruritus prevalence was higher in the HF group (44/74, 59.5%) compared with the non-HF group (6/26, 23.1%; p=0.001), and median VAS itchiness scores were significantly higher (15.0 mm vs. 0.0 mm, p<0.001). Multivariate analysis revealed that HF diagnosis (OR 7.15, 95% CI 1.17-43.69), forearm sebum content (OR 0.07, 95% CI 0.01-0.52), and use of moisturizers (OR 7.09, 95% CI 1.35-37.4) were independently associated with pruritus, while moisture content, antihistamine use, age, and BMI were not.

Conclusion: Older adults with HF exhibited a higher prevalence and severity of pruritus than non-HF older adults. Reduced sebum content, rather than reduced hydration, was independently associated with pruritus, suggesting that lipid deficiency played a key role in its pathogenesis. These findings underscore the importance of addressing local skin factors in addition to systemic contributors. Incorporating lipid-replenishing emollients into home and community care may represent a practical strategy to reduce pruritus and improve the quality of life in older adults with HF.

## Introduction

The increasing proportion of older adults, particularly in Japan, has been accompanied by a rising prevalence of heart failure (HF), mainly attributable to age-related hypertension and valvular disease [1-3]. Older adults with HF often report distressing symptoms, including dry skin and pruritus, that can affect quality of life (QOL) [4-7]. Even among those without HF, the severity, frequency, and chronicity of pruritus are known to significantly affect QOL [8].

In HF, physiological and treatment-related factors, such as reduced cardiac output, fluid restriction, and chronic diuretic use, may compromise the skin barrier function by lowering the stratum corneum moisture and sebum, more so than in older adults without HF. These mechanisms may contribute to the high prevalence of pruritus observed in HF populations. Previous studies have reported that pruritus occurs in 17-40% of patients with HF [6,7,9], with prevalence varying according to disease stage and study population [10]. Patients with CHF have described pruritus as a disturbing symptom that warrants clinical attention [6]. Moreover, pruritus has been associated with insomnia and reduced QOL, and insomnia has been identified as an independent predictor of adverse cardiac events in patients with HF [11].

In the home care setting, research on HF has focused on preventing rehospitalization and managing disease progression through circulatory control [12-20]. However, few studies have addressed chronic symptoms such as pruritus, which may impair the QOL of older adults with HF living at home. One study in HF outpatients reported that 64 of 112 (57.1%) experienced pruritus [21]. Although this finding underscores the relevance of skin care for pruritus in HF management, it remains unclear whether such a high incidence of pruritus was characteristic of HF or was equally common among older adults without HF.

This study aimed to compare stratum corneum moisture, sebum content, and the frequency and severity of pruritus between older adults with and without HF and identify skin-related characteristics to guide preventive skin-care strategies in home-care nursing.

## Materials and methods

Study design and period 

This prospective cross-sectional study was conducted at two hospitals and community settings in Japan between October 2021 and December 2022. To account for seasonal variation, measurements were performed during two periods (October-December and March-April).

Participants 

Eligible participants were older adults with HF (HF group) or community-dwelling older adults without HF (non-HF group) who provided written informed consent. For the non-HF group, eligibility required no self-reported history or diagnosis of HF and no current treatment for HF, confirmed by interview. Exclusion criteria for both groups were dementia or suspected dementia, difficulty in verbal communication, serious skin disorders, use of a ventricular-assist device, hemodialysis, chemotherapy, liver cancer, or cirrhosis. Among the analyzed participants, for the HF group, we recruited outpatients aged ≥20 years diagnosed with HF, with either a history of HF hospitalization or an ejection fraction < 40%. After applying the exclusion criteria, 112 outpatients were enrolled. Of these, 38 patients under age 65 were further excluded, leaving 74 older adults with HF. For the non-HF group, we recruited 42 community-dwelling older adults from monthly local senior clubs. After excluding 16 under the age of 65, 26 older adults without HF (aged ≥65 years) remained.

Measurements 

Skin Moisture and Sebum Content

Skin moisture (water) and sebum (oil) were measured on the anterior lower leg, specifically at the mid-point of the tibia, a region known to be prone to dryness. Measurements were performed with the participant seated and the leg exposed to the air for 15 minutes prior to measurement to allow acclimatization to the environmental conditions. Each measurement was performed three times, and the average value was used for analysis. A non-invasive device, the Derma Unit SSC3 (Courage & Khazaka, Germany), was used according to the device guidelines [22]. 

Environmental Conditions

The environmental conditions during measurements were as follows: 

HF group: Temperature (mean ± SD: 24.0 ± 1.4°C, median: 24.3°C, range: 20.1-26.5°C); humidity (mean: 43.2 ± 9.9%, median: 43.9%, range: 26.6-71.4%) 

Non-HF group: Temperature (mean ± SD: 20.1 ± 1.9°C, median: 19.9°C, range: 16.0-23.1°C); humidity (mean: 54.7 ± 5.0%, median: 54.3%, range: 47.4-66.3%). 

Assessment of Pruritus

The following information was collected from all participants: (i) Demographic and Clinical Information: Gender, age, weight, height, employment status, use of moisturizers, and antihistamine medication; (ii) Assessment of Pruritus: The presence and severity of pruritus were evaluated using the Japanese version of the 5-D Itch Scale [23,24] and a visual analog scale (VAS), referring to symptoms experienced during the preceding two weeks.

Statistical analysis

All analyses were performed using IBM SPSS Statistics for Windows, Version 28 (Released 2021; IBM Corp., Armonk, New York, United States). Continuous variables are presented as median with interquartile range (IQR), and categorical variables as frequency and percentage. Comparisons between the HF group and the non-HF group were conducted using the Mann-Whitney U test for continuous variables and the chi-square test or Fisher’s exact test for categorical variables, as appropriate.

Multivariate logistic regression analysis was conducted to identify factors associated with the presence of pruritus. Variables with p<0.10 in univariate analysis or considered clinically relevant were included. Multicollinearity was assessed using the variance inflation factor (VIF), and variables with VIF ≥10 were excluded. Odds ratios (ORs) and 95% confidence intervals (CIs) were calculated. Statistical significance was set at p <0.05.

As this was an exploratory cross-sectional study, all eligible participants during the study period were included, and therefore, no a priori sample size calculation was performed.

Ethical considerations

This study was conducted in accordance with the principles of the Declaration of Helsinki and was approved by the Institutional Ethics Committee (Approval No. R3-25). All participants were informed of the study’s purpose, methods, voluntary nature of participation, anonymity, protection of personal information, and the possibility of publication of the results. Written informed consent was obtained from all participants.

## Results

Participant characteristics and skin parameters

The participant characteristics and skin parameters by HF status are shown in Table 1. There were no significant differences between the HF and non-HF groups in terms of age, gender distribution, BMI, or occupation.

**Table 1:** Participant Characteristics and Skin Parameters With and Without HF p<0.05 Note: In the HF group, NYHA functional classification was as follows: Class Ⅰ (n=34, 45.9%), Class Ⅱ (n=37, 50.0%), Class Ⅲ (n=3, 4.1%) HF: Heart failure

Characteristics	Objective	HF	Non-HF	p-value
Sex	Male (%), Female (%)	53 (71.6), 21 (28.4)	18 (69.2), 8(30.8)	0.817
Age (years)	Median (IQR)	74.0 (70.0-78.0)	74.0 (71.0-77.5)	0.735
BMI (kg/m^2^)	Median (IQR)	22.3 (20.5-25.6)	23.8 (21.6-24.9)	0.963
Occupation	Yes (%)	21 (28.4)	12 (46.2)	0.097
Forearm skin moisture (a.u.)	Median (IQR)	34.4 (28.8-41.7)	43.7 (40.3-50.7)	<0.001
Forearm sebum (μg/cm^2^)	Median (IQR)	0.3 (0.0-0.6)	0.0 (0.0-0.5)	0.430
Forearm PH	Median (IQR)	5.7 (5.4-6.0)	5.5 (5.1-5.7)	0.063
Lower leg skin moisture (a.u.)	Median (IQR)	28.1 (22.0-37.9)	39.5 (34.9-42.1)	<0.001
Lower leg sebum (μg/cm^2^)	Median (IQR)	0.3 (0.0-0.7)	0.0 (0.0-0.3)	0.201
Lower leg PH	Median (IQR)	5.7 (5.5-6.1)	5.6 (5.2-5.7)	0.015
Itchy	Yes (%)	44 (59.5)	6 (23.1)	0.001
Pruritus VAS (mm)	Median (IQR)	15.0 (0.0-38.0)	0.0 (0.0-3.5)	<0.001
5D (itching over the past two weeks)	Not present (%)	30 (40.5)	20 (76.9)	0.025
5D (itching over the past two weeks)	Mild (%)	25 (33.8)	5 (19.2)	
5D (itching over the past two weeks)	Moderate (%)	15 (20.3)	1 (3.8)	
5D (itching over the past two weeks)	Severe (%)	3 (4.1)	0 (0.0)	
5D (itching over the past two weeks)	Unbearable (%)	1 (1.4)	0 (0.0)	
Use of antihistamine	Yes (%)	7 (9.5)	1 (3.8)	0.561
Use of moisturizer	Yes (%)	13 (17.6)	9 (34.6)	0.071

Skin moisture and sebum content

Both forearm and lower leg skin moisture levels were significantly lower in the HF group compared to the non-HF group (forearm: 34.4 [28.8-41.7] vs. 43.7 [40.3-50.7], p < 0.001; lower leg: 28.1 [22.0-37.9] vs. 39.5 [34.9-42.1], p <0.001). These differences may have been influenced by variations in measurement conditions, as the HF group was examined under higher temperatures and lower humidity. The pH of the lower leg was also significantly lower in the HF group (5.7 [5.5-6.1] vs. 5.6 [5.2-5.7], p = 0.015). No significant differences were found in skin oil content on either site.

Pruritus

The prevalence of pruritus was significantly higher in the HF group than in the non-HF group (44/74, 59.5% vs. 6/26, 23.1%; p =0.001). The median VAS itch score was also significantly higher in the HF group (15.0 [0.0-38.0]) compared to the non-HF group (0.0 [0.0-0.3]; p<0.001). Over the past two weeks, moderate to severe or unbearable itching was observed only in the HF group. The proportion of participants reporting no itching was higher in the non-HF group (20/26, 76.9%) compared to the HF group (30/74, 40.5%; p = 0.025).

Medication

There were no significant differences between groups in the use of antihistamines. The use of moisturizers tended to be higher in the non-HF group, but this difference did not reach statistical significance (p =0.071).

Factors associated with pruritus 

Table 2 presents the results of the multivariate logistic regression analysis. The presence of HF was significantly associated with pruritus (OR: 7.15, 95% CI: 1.17-43.7, p =0.033). Forearm sebum was also significantly associated with an increased risk of pruritus (OR: 0.07, 95% CI: 0.01-0.52, p =0.009). In addition, the use of moisturizers emerged as an independent factor associated with pruritus (OR: 7.09, 95% CI: 1.35-37.4, p =0.021).

**Table 2:** Multivariate Logistic Regression Analysis of Factors Associated with Pruritus p<0.05, CI: confidence interval

Variable	Comparison	Odds ratio	95% CI	p-value
HF	(presence vs absence)	7.15	1.17 - 43.69	0.033
Forearm sebum	(per unit increase)	0.07	0.01-0.52	0.009
Use of moisturizer	(yes vs no)	7.09	1.35 - 37.4	0.021
Use of antihistamine	(yes vs no)	7.58	0.53 - 107.97	0.135
Occupation	(yes vs no)	0.30	0.08 - 1.17	0.084
Forearm skin moisture	(per unit increase)	1.03	0.96 - 1.11	0.418
Age	(per year)	1.07	0.96 - 1.20	0.218
BMI	(per unit increase)	1.04	0.89- 1.21	0.629

Other variables, including antihistamine use, occupation, forearm skin moisture, age, and BMI, were not significantly associated with pruritus in the multivariate model.

## Discussion

This study found that older adults with HF had a higher prevalence and severity of pruritus compared with non-HF participants. While skin water content was lower in the HF group, this difference may reflect the measurement condition. By contrast, lower forearm sebum content was independently associated with pruritus, suggesting that lipid deficiency rather than reduced hydration may play a key role in symptom development.

Previous studies have attributed pruritus in HF to systemic factors such as renal dysfunction, hepatic congestion, and inflammation. Although these parameters were not examined in the present study, our results highlight the importance of local skin conditions-particularly diminished sebum-in the pathogenesis of pruritus. Research on CKD-associated pruritus emphasizes the contribution of impaired renal function and systemic inflammation [25]. Similarly, hepatic congestion and cholestasis, common in advanced HF, have been recognized as potential triggers [26,27]. In addition, autonomic imbalance and thermoregulatory dysfunction in HF may facilitate inflammatory and neuro-immune pathways leading to itch [28,29]. Consistently, a prevalence study suggested that patients with CHF experience pruritus more frequently than the general population [6].

The findings of this study that reduced sebum content was independently associated with pruritus in older adults with HF may be explained by alterations in the skin lipid barrier. Lipidomics studies in older adults have shown that changes in skin surface lipid composition, particularly ceramides, correlate with pruritus severity [30]. Recent reviews further emphasize that ceramides and other stratum corneum lipids are essential for maintaining barrier integrity and regulating inflammation and itch [31]. Consistent with this, our results suggest that lipid deficiency, rather than reduced hydration alone, may play an important role in itch pathogenesis among older adults with HF.

In addition, disruption of the skin barrier is known to activate inflammatory and neuro-immune pathways that contribute to chronic itch [32]. In HF, systemic factors such as diuretic use, congestion, and comorbid organ dysfunction may exacerbate this barrier impairment, creating a multifactorial pathway to pruritus.

From a practical perspective, implementation of effective skin care remains challenging. A recent study identified barriers such as heavy workload, challenges in caring for older adults with cognitive impairment, and unrealistic family expectations, whereas knowledge of skin hygiene care served as a facilitator [33]. Similar barriers may exist in home care for older adults with HF, where sustained benefits depend not only on professional input but also on daily self-care by patients and caregivers.

These findings have important implications for community and home-based HF management. Skin care strategies should prioritize lipid-replenishing emollients, such as formulations containing ceramides, cholesterol, and free fatty acids, rather than water-only moisturizers. Education and support for patients and caregivers are essential to overcome practical barriers and to ensure continuity of care. Integrating systematic itch screening with lipid-focused emollient use in home care settings may therefore represent a practical approach to reduce pruritus and improve QOL in older adults with HF.

Limitations 

This study has several limitations. First, the sample size was small, and the wide CIs in the regression analysis indicate limited precision. Moreover, as this was an exploratory study, no a priori sample size calculation was performed. This limits the ability to assess whether the study was adequately powered to detect clinically meaningful differences. Second, measurement conditions differed between groups, which may have confounded water content values. Third, systemic parameters such as renal and hepatic function, inflammatory markers, and diuretic dosage were not assessed, precluding further exploration of systemic contributions. In particular, the absence of comparative blood data between the HF and non-HF groups limits our ability to evaluate systemic confounders that may influence the prevalence and severity of pruritus. Finally, moisturizer use was included in the regression model, although its association with pruritus likely reflects reverse causality.

Future directions 

Future studies should employ standardized measurement conditions, larger sample sizes, and a comprehensive assessment of systemic factors. In addition, intervention studies are warranted to evaluate whether lipid-replenishing emollients, combined with patient and caregiver education, can effectively reduce pruritus and improve QOL in older adults with HF, particularly in community and home care settings.

## Conclusions

This study found that older adults with HF experienced a higher prevalence and severity of pruritus compared with non-HF participants. Reduced sebum content, rather than reduced hydration, was independently associated with pruritus, suggesting that lipid deficiency plays an important role in its pathogenesis. These findings highlight the need to consider local skin factors in addition to systemic contributors when managing pruritus in HF. Incorporating lipid-replenishing emollients into routine care, particularly in community and home settings, could provide a practical strategy to reduce symptoms and improve QOL.
